# Interorgan Crosstalk Contributing to *β*-Cell Dysfunction

**DOI:** 10.1155/2017/3605178

**Published:** 2017-01-12

**Authors:** Katsuya Tanabe, Kikuko Amo-Shiinoki, Masayuki Hatanaka, Yukio Tanizawa

**Affiliations:** Division of Endocrinology, Metabolism, Hematological Science and Therapeutics, Yamaguchi University Graduate School of Medicine, Ube, Yamaguchi, Japan

## Abstract

Type 2 diabetes mellitus (T2DM) results from pancreatic *β*-cell failure in the setting of insulin resistance. In the early stages of this disease, pancreatic *β*-cells meet increased insulin demand by both enhancing insulin-secretory capacity and increasing *β*-cell mass. As the disease progresses, *β*-cells fail to maintain these compensatory responses. This involves both extrinsic signals and mediators intrinsic to *β*-cells, which adversely affect *β*-cells by impairing insulin secretion, decreasing proliferative capacities, and ultimately causing apoptosis. In recent years, it has increasingly been recognized that changes in circulating levels of various factors from other organs play roles in *β*-cell dysfunction and cellular loss. In this review, we discuss current knowledge of interorgan communications underlying *β*-cell failure during the progression of T2DM.

## 1. Introduction

Type 2 diabetes mellitus (T2DM) is a complex multifactorial disorder characterized by both insulin resistance and defects in pancreatic *β*-cell function. An important feature of this disease in the early stage are the physiological responses of *β*-cells as they adapt, via both enhanced function and increased morphological mass, to the increased insulin demand imposed by insulin resistance [1, 2]. However, as the disease progresses, the chronically increased workload on remaining *β*-cells results in their failure, ultimately leading to hyperglycemia [3]. Based on numerous experiments in rodent models and human subjects, it is believed that the failure of *β*-cells to increase mass and function is central in T2DM [3–6] and that both extrinsic signals and mediators intrinsic to *β*-cells are involved in the development of *β*-cell failure.

Individual *β*-cells can sense a multitude of signals that are integrated into physiological *β*-cell responses to metabolic demand. Particularly, nutrients are essential for the maintenance of *β*-cell function and mass [7]. However, in the diabetic milieu, chronic excess of nutrients such as glucose, free fatty acids (FFAs), and lipid intermediates synergistically induces deleterious effects on both *β*-cell mass and function and creates a vicious cycle that contributes to the progressive loss of functional *β*-cell mass (glucolipotoxicity) [8]. In addition to overnutrition, changes in the levels of various circulating factors derived from peripheral tissues such as adipocytes, the skeletal system, and various immune cells, not only constitute a significant link between obesity and insulin resistance but also adversely affect *β*-cells by impairing their functions and limiting cell mass. In this review, we will discuss some of the major mechanisms underlying the concomitant effects of interorgan crosstalk associated with *β*-cells and how insulin resistance negatively impacts both the function and mass of *β*-cells.

## 2. Adipocyte to *β*-Cell Crosstalk Mediated by the Adipokines

Insulin resistance resulting from obesity is associated with a particular milieu of circulating factors in the plasma, any of which could signal *β*-cells to fail to adapt to increased insulin demand. The role of adipose tissue as an active endocrine organ rather than simply an energy storage depot is now well appreciated, and plasma levels of adipocyte-secreted hormones (adipokines) are altered in obese subjects [9]. Obese adipocytes, which become hypertrophic as lipid contents increase, secrete less adiponectin and more leptin and proinflammatory cytokines [10, 11]. The release of FFAs, which have been shown to activate inflammatory signaling, may also be increased in obesity as a result of activated lipolysis. Although modified adipokines were initially recognized as exerting effects on the hypothalamus and peripheral tissues as an important link between obesity and insulin resistance, a more detailed understanding of the interactions between these factors and *β*-cells has recently emerged [12, 13].

In obese individuals, hyperphagia is associated with high levels of the adipocyte-derived hormone leptin [14]. The hypothalamic actions of leptin are relatively well characterized, though leptin can also exert peripheral actions independently of its effects on the hypothalamus [11, 14]. The long form of the leptin receptor (ObRb) that is capable of intracellular signaling is expressed in *β*-cells, and exogenous leptin inhibits insulin production and secretion from human islets [15–19], suggesting a direct action on *β*-cells. Regarding the in vivo relevance of leptin and *β*-cells, experiments on mice with conditional ablation of the leptin receptor (ObR) in *β*-cells revealed that leptin plays roles in regulating *β*-cell function and mass. Mice with a floxed ObR were crossed with mice expressing Cre recombinase under the control of the Pdx1 promoter, which is not expressed in the hypothalamus. *β*-Cell specific deletion of the ObR gene resulted in improved glucose tolerance and enhanced insulin secretion [20]. A 2-fold increase in *β*-cell mass in the absence of insulin resistance was documented, suggesting that leptin negatively affects *β*-cell mass. In the setting of high fat diet-induced obesity, however, *β*-cell specific loss of the leptin receptor worsened glucose tolerance, impairing both insulin secretion and expansion of *β*-cell mass [20]. These data suggest complicated leptin actions. Whereas leptin has inhibitory effects on *β*-cell function and expansion under normal metabolic conditions [20], the high plasma leptin levels accompanying increased adiposity could play a role in *β*-cell adaptation in the setting of high fat diet-induced obesity in mice [20]. However, the precise in vivo mechanisms of leptin action on *β*-cells have yet to be elucidated. A more recent study using a different line expressing Cre in *β*-cells while avoiding neuronal Cre expression raised the possibility that the in vivo effects of leptin may not be mediated through its receptor on *β*-cells, suggesting instead indirect leptin actions on *β*-cells [23]. In this regard, leptin acts on *β*-cells, at least partly, by modulating the bioactivity of osteocalcin, an osteoblast-secreted hormone (outlined in Section 3 of the adipocyte-brain-bone-*β* cell axis). On the other hand, the leptin administration in animal models of T1DM prevents hyperglycemia and ketoacidosis without the restoration of insulin deficiency. The suppression of the glucagon actions in liver and the activation of leptin receptors in the central nervous system underlie the antidiabetic actions of leptin in the context of T1DM [24, 25].

Adiponectin, another adipocyte-derived hormone, of which the circulating level correlates negatively with obesity and T2D, facilitates *β*-cell regeneration in mice with STZ-induced *β*-cell ablation [26]. Additionally, this hormone was recently shown to protect *β*-cells from the harmful effects of FFA [27]. Although how adiponectin exerts protective effects on *β*-cells remains unknown, it is likely that a paucity of circulating adiponectin relative to leptin and proinflammatory cytokines would be an important factor in the overall effects on *β*-cells of the altered adipokine profiles that correlate with increased adiposity [28].

Excess FFA from obese adipose tissue contributes to marked elevations of circulating FFAs. Clinically, high FFA levels, particularly saturated fatty acids, are an independent predictor of future T2DM [29]. Palmitate is the most abundant saturated FFA in blood, and the deleterious effects of palmitate, collectively termed as “lipotoxicity,” on *β*-cells are well documented [30]. In vitro studies using isolated islets and clonal *β*-cells have shown that *β*-cell lipotoxicity is directly induced by palmitate, at least in part via pathways primarily involving endoplasmic reticulum (ER) stress and reactive oxygen species (ROS) [31–34]. More recent studies have shown that FFA modulates inflammation within islets [35]. Palmitate is capable of TLR4 activation in *β*-cells. This was observed to be followed by induction of chemokines (e.g., MCP1/CCL2) and inflammatory cytokines (e.g., IL-1*β*) and their release from *β*-cells, resulting in local islet inflammation mediated by interactions between M1 macrophages and *β*-cells [35] (outlined in Section 7 of the interplay between immune cells and *β*-cells).

## 3. Bone to *β*-Cell Crosstalk

Bone has emerged as an endocrine organ regulating glucose and energy metabolism, suggesting that bone is active in the processes of regulating fuel consumption to adapt to locomotive activity [36, 37]. The osteoblast-specific secreted molecule osteocalcin enhances insulin secretion, insulin sensitivity and energy, expenditure [38, 39]. Osteocalcin, one of the most abundant components of the bone extracellular matrix, is synthesized and secreted by osteoblasts. Osteocalcin undergoes posttranslational carboxylation on three glutamic residues (located at positions 17, 21, and 24) in glutamic acid, which is involved in calcium and hydroxyapatite binding and deposition in the extracellular matrix of bone. In contrast, undercarboxylated osteocalcin has a low affinity for hydroxyapatite and is more easily released into the circulation, allowing it to reach target tissues and exert its endocrine functions. Mice lacking osteocalcin exhibit glucose intolerance resulting from the coexistence of impaired insulin secretion and insulin resistance [38, 40]. Conversely, augmentation of osteocalcin's bioactivity due to inactivation of osteotesticular protein tyrosine phosphatase (OST-PTP) encoded by a gene termed* Esp* in mice enhances both insulin secretion and *β*-cell expansion [38]. Recently, Gprc6a, a GPRC, was identified as a specific receptor for osteocalcin and is known to be essential for osteocalcin functions in *β*-cells. Gprc6a is expressed in Leydig cells of the testes and in pancreatic *β*-cells. Genetic evidence obtained in mice indicates that Gprc6a is needed for osteocalcin regulation of insulin secretion and pancreatic *β*-cell proliferation [41].

**Figure 1:**
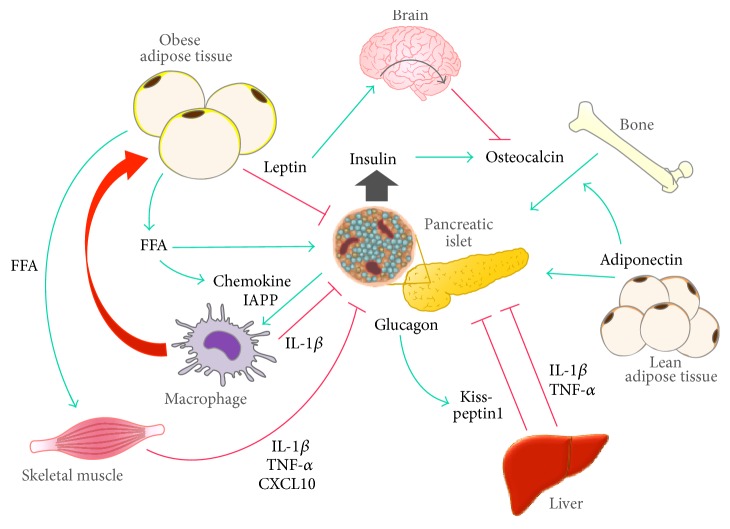
The interorgan crosstalk involved in *β*-cell failure. Representative pathways from metabolic organs involved in a reduction of functional *β*-cell mass are illustrated. Although adiponectin, preferentially secreted from lean adipose tissue, may have protective effects on *β*-cells, leptin, which is secreted more from obese adipose tissue, negatively impacts *β*-cell function and mass via direct and indirect pathways. Leptin suppresses the bioactivity of osteocalcin, which is essential for *β*-cell function and expansion, through the modulation of sympathetic tone signals delivered to osteoblasts, creating a feed-forward interplay among adipose tissue, the brain, bone, and *β*-cells. Conversely, insulin enhances osteocalcin bioactivity through the activation of osteoclastic bone resorption. Meanwhile, excess FFA spillover from obese adipose tissue induces insulin resistance in insulin sensitive organs such as muscle and liver, resulting in overload of *β*-cells by excess insulin demand. Additionally, skeletal muscle and liver might exert detrimental effects of *β*-cell function by secreting proinflammatory cytokines and chemokines in this setting. Glucagon promotes Kisspeptin 1 production in hepatocyte, which mediates an alternative pathway from liver to *β*-cells. Furthermore, FFA induces the production of chemokines in *β*-cells, recruiting M1 macrophages into islets. In the diabetic milieu, hyperglycemia and IAPP derived from *β*-cells synergistically promote inflammatory responses through the promotion of IL-1*β* biosynthesis in M1 macrophages.

Although several lines of genetic and biochemical evidence clearly demonstrate direct effects of undercarboxylated osteocalcin on *β*-cells, the *β*-cell-derived signal regulating the bioactivity of osteocalcin has recently been identified. Osteoblasts express insulin receptors, and insulin signaling in osteoblasts would presumably be necessary for whole body glucose homeostasis through regulating osteocalcin bioactivity in mice [40]. Additionally, it was revealed that OST-PTP, a tyrosine phosphatase negatively regulating the metabolic actions of osteocalcin, attenuates insulin signaling through dephosphorylation of the insulin receptors in osteoblasts, providing the molecular mechanism by which OST-PTP impairs osteocalcin bioactivity [40]. Mice lacking insulin receptors specifically in osteoblasts exhibited a decrease in circulating levels of the active form of osteocalcin, glucose intolerance, impaired insulin secretion, and insulin resistance [40]. A complicated mechanism, operating at the molecular level, has been proposed to underlie insulin-mediated osteocalcin activation. Insulin signaling in osteoblasts facilitates osteoclastic bone resorption via the inhibition of osteoblast expression of osteoprotegerin, an inhibitor of osteoclast differentiation. This process may allow acidification of the bone extracellular matrix. Indeed, a low pH is the only known means of decarboxylating proteins outside of cells. Thereby, low pH associated with bone resorption can contribute to decarboxylating and activating osteocalcin. As noted above, accumulating lines of genetic and biochemical evidence illustrate that a feed-forward interplay between *β*-cells, osteoblasts, and osteoclasts regulates *β*-cell function and whole body glucose metabolism [42] (Figure 1).

## 4. Adipocyte-Brain-Bone-*β* Cell Axis

The adipocyte-derived hormone leptin negatively regulates bone formation by modulating sympathetic innervation of osteoblasts [43]. As discussed in Section 2, leptin negatively regulates *β*-cell function and mass as well. However, some of the endocrine actions of leptin on *β*-cells may not be mediated via its receptors on *β*-cells [20, 23, 44]. In this context, the fact that leptin regulates bone metabolism implies that bone may exert feedback control on *β*-cell function. Indeed, leptin negatively regulates osteocalcin bioactivity via increasing sympathetic tone in mice. Thereby, leptin actions on *β*-cells, at least partly, might be mediated by an adipocyte-brain-bone interplay [45–48]. In addition to leptin, adiponectin, another adipose-derived hormone, also participates in the regulation of bone formation and osteocalcin production. However, adiponectin reduces sympathetic tone via its hypothalamic actions and counteracts the effects of leptin on bone formation [49]. Collectively, several types of experimental evidence obtained in mice illustrate the feed-forward interplay effects among adipocytes, the brain, bone, and *β*-cells, which are involved in glucose homeostasis and *β*-cell function (Figure 1).

## 5. Liver to *β*-Cell Crosstalk

Obesity and inflammation are highly integrated processes in the pathogenesis of insulin resistance, diabetes, dyslipidemia, and nonalcoholic fatty liver disease (NAFLD) [50, 51]. In liver with NAFLD, Kupffer cells, which are liver resident macrophages of the reticuloendothelial system, play a similar role to M1 macrophage and, thereby, secrete inflammatory cytokines, which contribute to worsening local or systemic inflammation and, in turn, perturb metabolic homeostasis [52–54]. In this regard, whereas there is the hypothesis that the liver insulin resistance stimulates *β*-cell replication [55, 56], liver with NAFLD plays a critical role in the progression of *β*-cell failure by augmenting *β*-cell workload and modulating islet inflammation (Figure 1).

Hyperglucagonemia is a common feature of T2DM, which causes an increase in hepatic glucose production. It has been recently reported that glucagon stimulates Kisspeptin 1 production through PKA signaling in hepatocyte and that liver derived Kisspeptin1 negatively impacts *β*-cell function in mouse models of insulin resistance [57]. Pancreatic *β* cells abundantly express Kisspeptin 1 receptor, which inhibits cAMP production and thereby inhibits insulin secretion [57]. Importantly, knockdown of Kisspeptin 1 in liver ameliorates glucose tolerance and increases GSIS in the mice fed on high fat diet and the mice with leptin receptor deficiency [57]. Taken together, these experimental evidences obtained in mice illustrate a novel endocrine circuit among *α*-cells, liver, and *β*-cells, which contributes to *β*-cell dysfunction (Figure 1).

## 6. Muscle to *β*-Cell Crosstalk

T2DM is associated with physiological changes in skeletal muscle. Skeletal muscle is the largest organ in nonobese subjects and a major site of insulin- and exercise-mediated glucose disposal. Thereby, it appears plausible that the muscle might interact with the islets and modulate insulin secretion for appropriate peripheral glucose utilization. An early study has revealed that muscle-specific deletion of PGC1*α* causes impaired glucose tolerance in the mice fed a high fat diet not via a decrease in peripheral insulin sensitivity but rather via impaired *β*-cell function, demonstrating skeletal muscle to *β*-cell crosstalk [58]. A possible mediator of this crosstalk is the IL-6, expression of which is increased in muscle-specific PGC1*α* knockout mice and which can inhibit glucose-stimulated insulin secretion in isolated islets [58]. However, another study proposed the opposite hypothesis that whole body IL-6 knockout mice fed a high fat diet show insulin-secretory defects, uncovering a role for IL-6 in *β*-cell compensation for insulin resistance [59]. Further, IL-6 regulates expansion of *α*-cell mass in culture and in vivo [59]. More recently, a role for IL-6 in a skeletal muscle-enteropancreatic circuit has been identified in mice subjected to exercise [60]. IL-6 produced by skeletal muscle in an exercise setting was found to promote glucagon-like peptide-1 (GLP-1) secretion from L-cells in the intestine and to further improve *β*-cell function by increasing islet GLP-1 through a modulation of posttranslational processing of proglucagon to favor the production of GLP-1 rather than glucagon, leading to improved glucose tolerance [60]. IL-6 is mostly secreted in response to muscle contraction and plays a critical role in the metabolic adaptation to exercise [61]. In this regard, it is conceivable that exercise not only alters insulin sensitivity in skeletal muscle but also improves *β*-cell function. However, the endocrine role of IL-6 in metabolism is yet to be fully understood, because the chronic effects of IL-6 remain controversial [59, 62, 63] whereas IL-6 elevated acutely with exercise might exert beneficial effects. Furthermore, it has been recognized that skeletal muscle produces alternative myotube-derived cytokines (“myokines”) with different profiles depending on insulin sensitivities. Insulin-resistant muscle contributes to proinflammatory milieu associated with impaired *β*-cell function. Recent in vitro studies suggested that insulin-resistant skeletal muscle affects *β*-cell function by secreting myokines with proinflammatory profiles, including IL-1*β*, TNF-*α*, and C-X-C motif ligand 10 (CXCL-10) [64, 65] (Figure 1).

## 7. Interplay between Immune Cells and *β*-Cells

As noted so far, obesity and T2DM are associated with chronic inflammation [66–68]. Islet inflammation has increasingly been demonstrated in T2DM subjects based on histological characteristics including amyloid deposition [69], immune cell infiltration [70], and *β*-cell fibrosis [71]. This suggests that inflammation is also involved in the development of *β*-cell failure. Although inflammation can be triggered by metabolic signals, how overnutrition and obesity initiate and sustain inflammation in islets has yet to be fully characterized. In response to a glucolipotoxic microenvironment, *β*-cells are very likely affected by the contributions of proinflammatory factors (e.g., IL-1*β*) derived from the *β*-cells themselves and from recruited immune cells including macrophages [72–75]. *β*-Cells are capable of producing chemokines (e.g., MCP1/CCL2) in the presence of high FFA levels, and hyperglycemia forces *β*-cells to produce islet amyloid polypeptide (IAPP) [35, 75–77]. In response to chemokines derived from *β*-cells, bone marrow derived M1-type macrophages infiltrate islets. Indeed, pharmacological blockade of the accumulation of M1 macrophages protects *β*-cells from the detrimental effects of palmitate, indicating the causal involvement of M1 macrophages [35]. In this context, the T2DM milieu may induce *β*-cell production of chemokines that promote M1 macrophage infiltration of islets. Furthermore, high levels of glucose activate NLRP3-dependent inflammasomes in islet resident macrophages, resulting in IL-1*β* processing and production [76, 77]. High glucose-mediated inflammasome activation is, at least in part, induced by a soluble oligomer of IAPP and ROS [77, 78]. Whereas low concentrations of IL-1*β* may enhance *β*-cell survival and function [79], persistent abundant production of IL-1*β* by M1 macrophages promotes *β*-cell dysfunction and exerts proapoptotic effects [80–85], and the secretion of chemokines from *β*-cells and cytokines from M1 macrophages then forms a vicious cycle that accelerates islet inflammation. Consistently, M1 macrophage accumulation within islets appears to contribute to *β*-cell dysfunction in mouse models of T2DM [35]. Therefore, the activation of inflammatory processes mediated by the interplay between macrophages and *β*-cells is an important factor in *β*-cell failure in the setting of T2DM.

Finally, the contribution of islet inflammation to *β*-cell failure in T2DM is further supported by both in vitro and in vivo studies employing pharmacological blockade of IL-1*β* signaling. For instance, an antagonist for IL-1 receptors, which are shared by IL-1*α* and IL-*β*, protects islets from the detrimental effects of glucotoxicity [86]. A clinical study using IL-1 receptor antagonist showed improved insulin secretion and a reduction in the proportion of proinsulin to insulin secreted in patients with T2DM [87]. More recently, gevokizumab, a recombinant humanized monoclonal antibody that neutralizes IL-1*β* and preserves IL-1*α* signaling, has been tested for its therapeutic impact in subjects with T2DM [88]. In this trial, an intermediate dose (0.03–0.1 mg/kg) of gevokizumab significantly improved glycemic control and C-peptide secretion. Interestingly, a high dose (>0.3 mg/kg) failed to exert antidiabetic effects. This observation may suggest a clinical relevance of the notion that a low concentration of IL-1*β* is rather beneficial for *β*-cells. Taken together, these studies illustrate the novel therapeutic concept that modulating the immune system can prevent *β*-cell failure and, thereby, can slow or even prevent the development of T2DM.

## 8. Gut to *β*-Cell Crosstalk

The incretin hormones glucose-dependent insulinotropic peptide (GIP) and glucagon-like peptide-1 (GLP-1) are secreted postprandially and act as circulating factors enabling the body to respond appropriately to food-derived elevations of blood nutrient concentrations. This is a significant physiological mechanism to maintain whole body glucose homeostasis, as costimulation of pancreatic *β*-cells by GIP and GLP-1 approximately doubles the amount of insulin released in response to an elevation in blood glucose concentrations. Following the discovery that the insulinotropic effect of GLP-1 is preserved in most patients with T2DM [89], GLP-1 mimetics and inhibitors of GLP-1 degradation by dipeptidyl peptidase 4 (DPP4) have been developed and licensed for the treatment of T2DM [90]. On the other hand, one of the options offered for extreme obesity is gastric bypass surgery such as Roux-Y gastric bypass, which provides significant weight loss and ameliorates hyperglycemia and insulin resistance. The increasing evidences of elevated postprandial GLP-1 levels after Roux-Y gastric bypass surgery strongly suggest benefits of recruiting endogenous GLP-1 reserves as a not yet exploited treatment alternative [91].

## 9. Conclusion

Progressive loss of functional *β*-cell mass is central to the development and progression of T2DM. Despite clinical use of various glucose lowering agents, the existing therapies are limited to preventing the progression of *β*-cell failure in T2DM, with the possible exception of gastric bypass surgery [92]. Numerous extrinsic pathways and intrinsic mediators underlie decreased *β*-cell function and reduced *β*-cell mass, perhaps a consequence of processes that initially impaired the functions of individual *β*-cells. In the presence of insulin resistance and under glucolipotoxic conditions, various extracellular signals from other organs modulate cellular responses, such as those involved in fuel metabolism, ER, and oxidative stress, as well as activating proinflammatory cascades and, in turn, constituting a vicious feed-forward cycle that promotes impaired insulin secretion, apoptosis, and perhaps dedifferentiation [93]. From such a viewpoint, interorgan regulation may play a causative role in the development of T2DM, at least in part, by modulating the processes that render *β*-cells unable to respond to increased metabolic demand. However, it is clear that more studies are needed to obtain a complete picture of the molecular mechanisms underlying *β*-cell failure in the setting of T2DM and how we can prevent its progression. There are likely to be additional important signals involved in *β*-cell failure that will be revealed in future studies. Also, the following enduring issues must be addressed as we move forward: (1) How can we translationally understand the interorgan interplay demonstrated in experimental animal models in terms of human pathophysiology? (2) What is the dominant pathway among the different pathways at various disease stages? The challenges ahead will include identifying pathways that are most applicable, feasible, and, ultimately, effective for the treatment of T2DM.
